# Engineering the expression system for *Komagataella phaffii (Pichia pastoris)*: an attempt to develop a methanol-free expression system

**DOI:** 10.1093/femsyr/foz059

**Published:** 2019-08-13

**Authors:** Shinobu Takagi, Noriko Tsutsumi, Yuji Terui, XiangYu Kong, Hiroya Yurimoto, Yasuyoshi Sakai

**Affiliations:** 1 Novozymes Japan Ltd, CB-6 MTG, 1–3 Nakase, Mihama-ku, Chiba 261–8501, Japan; 2 Novozymes (China) Investment Co. Ltd, 14 Xinxi Road, Shangdi Zone, Haidian District, 100085 Beijing, China; 3 Division of Applied Life Sciences, Graduate School of Agriculture, Kyoto University, Kitashirakawa-Oiwake, Sakyo-ku, Kyoto 606–8502, Japan

**Keywords:** *Komagataella phaffii*, *Pichia pastoris*, *DAS1* promoter, *KpTRM1*, *PRM1*, *MXR1*, phytase

## Abstract

The construction of a methanol-free expression system of *Komagataella phaffii* (*Pichia pastoris*) was attempted by engineering a strong methanol-inducible *DAS1* promoter using *Citrobacter braakii* phytase production as a model case. Constitutive expression of *KpTRM1*, formerly *PRM1*—a positive transcription regulator for methanol-utilization (MUT) genes of *K. phaffii*,was demonstrated to produce phytase without addition of methanol, especially when a *DAS1* promoter was used but not an *AOX1* promoter. Another positive regulator, Mxr1p, did not have the same effect on the *DAS1* promoter, while it was more effective than KpTrmp1 on the *AOX1* promoter. Removing a potential upstream repression sequence (URS) and multiplying UAS1_DAS1_ in the *DAS1* promoter significantly enhanced the yield of *C. braakii* phytase with methanol-feeding, which surpassed the native *AOX1* promoter by 80%. However, multiplying UAS1_DAS1_ did not affect the yield of methanol-free expression by constitutive KpTrm1p. Another important region to enhance the effect of KpTrm1p under a methanol-free condition was identified in the *DAS1* promoter, and was termed ESP_DAS1_. Nevertheless, methanol-free phytase production using an engineered *DAS1* promoter outperformed phytase production with the *GAP* promoter by 25%. Difference in regulation by known transcription factors on the *AOX1* promoter and the *DAS1* promoter was also illustrated.

## Teaser

Engineering the methanol-inducible *DAS1* promoter to develop an efficient methanol-free expression system in *K. phaffii* (*P. pastoris*).

## ABBREVIATIONS

AOX: alcohol oxidase
DAS: dihydroxyacetone synthase
MUT: methanol-utilization
UAS: upstream activation sequence
URS: upstream repression sequence

## INTRODUCTION

Methylotrophic yeast such as *Komagataella phaffii* (formerly *Pichia pastoris*)*, Ogataea methanolica (P. methanolica*)*, O. polymorpha (Hansenula polymorpha)* and *Candida boidinii* possess a common methanol utilization (MUT) metabolic pathway (Hartner and Glieder 2006; Yurimoto and Sakai 2009)—including alcohol oxidase (AOX), dihydroxyacetone synthase (DAS) and formate dehydrogenase (FDH)—to grow with methanol as carbon and energy source. These MUT genes are highly induced in the presence of methanol, of which the induction mechanism has not been well understood. Heterologous protein expression systems have been established utilizing methanol-inducible promoters from MUT genes and are being used for production of biopharmaceutical proteins and industrial enzymes (Cereghino and Cregg 2000; Gellissen 2000; Macauley-Patrick *et al*. 2005; Ahmad *et al*. 2014; Yurimoto *et al*.^2000^).

Among methylotrophic yeasts, *K. phaffii* is by far the most utilized. A wide range of proteins from various origins—from bacteria to human—have been expressed in *K. phaffii*, especially using the *AOX1* promoter with methanol induction, at a yield from milligrams per liter of broth to over 20 g per liter of broth in high-cell-density fermentation (Cereghino and Cregg 2000; Gellissen 2000; Macauley-Patrick *et al*. 2005). However, methanol is a flammable chemical that can be hazardous to use at an industrial scale (Gellissen 2000; Ahmad *et al*. 2014). For industrial use, therefore, a methanol-free expression system is preferable. A strong constitutive promoter from the glyceraldehyde-3-phosphate dehydrogenase gene (*GAP*) that does not require methanol induction is also used in *K. phaffii* for protein production (Cereghino and Cregg 2000; Macauley-Patrick *et al*. 2005; Ahmad *et al*. 2014). However, protein yields using the *GAP* promoter reported so far are slightly inferior to the ones using the *AOX1* promoter with methanol. Moreover, unlike methanol inducible promoters, the *GAP* promoter is not controllable; therefore, it is not suitable for the production of proteins, which are toxic to the cells (Cereghino and Cregg 2000; Macauley-Patrick *et al*. 2005; Ahmad *et al*. 2014). Thus, the *AOX1* promoter with methanol induction is the most commonly used expression system for protein production in *K. phaffii* (Macauley-Patrick *et al*. 2005; Ahmad *et al*. 2014).

The regulation of the *AOX1* promoter consists of three steps (Vogl *et al*. 2018): i) Repression by nonmethanol carbon sources, such as glucose, glycerol and ethanol. The *AOX1* promoter is tightly repressed by the presence of such repressive carbon sources. ii) De-repression through the depletion of repressive carbon source. Once the repressive carbon source is depleted, the *AOX1* promoter is weakly induced. iii) Methanol induction. The *AOX1* promoter is fully induced in the presence of methanol. Transcription factors responsible for i) are negative regulators while the ones responsible for ii) or iii) are positive regulators.

Transcription factors recognize specific DNA sequences (*cis*-elements) in the promoter of a targeted gene and bind it through the DNA binding domain to regulate the transcription of the gene. The DNA binding domain is classified into three domains, namely, the zinc-containing domain, such as C_2_H_2_ zinc finger (Adr1 type) and Zn_2_Cys_6_ zinc cluster (Gal4 type); helix-turn-helix (Matα2 type); and the zipper-type domain (Gcn4 type) (Hahn and Young 2011). Activation or inactivation of the DNA binding of transcription factors could be controlled by other factors such as kinase, mediator and chaperon (Hahn and Young 2011, Traven, Jelicic and Sopta 2006). For instance, AMP-activated protein kinase Snf1 is essential for the DNA binding of Adr1p in *Saccharomyces cerevisiae*, which is a positive regulator for the catalytic pathway of nonfermentable sugar (de-repression) (Turcotte *et al*. 2010; Hahn and Young 2011). Snf1 kinase also controls the activity of glucose repressor Mig1p by exporting it from nuclear to cytoplasm, resulting in de-repression (Klein, Olsson and Nielsen 1998; Turcotte *et al*. 2010). In fact, Li *et al*. (2018) indicated that the AMPK/SNF1 pathway is likely to promote the activity of the *AOX1* promoter in *K. phaffii* via transcription factors and/or mediators.

Several transcription factors regulating MUT genes have been identified (Yurimoto, Oku and Sakai 2011; Vogl *et al*. 2018). *C. boidinii* has two positive regulators: Trm1p (Sasano *et al*. 2008) and Trm2p (Sasano *et al*. 2010)—the former is responsible for methanol induction and the latter likely responsible for de-repression, and one glucose repressor, Mig1p (Zhai, Yurimoto and Sakai 2012), has been identified. In *K. phaffii*, Mxr1p (Lin-Cereghino *et al*. 2006) of Adr1 type and Prm1p (Takagi *et al*. 2012; Wang *et al*. 2016b)/Trm1p (Sahu, Rao and Rangarajan 2014) and Mit1p (Wang *et al*. 2016b) of Gal4 type are known positive regulators for *AOX1* where the former is a regulator for de-repression and the latter is for methanol induction. Nrg1p (Wang *et al*. 2016a) is a glucose/glycerol repressor for *AOX1*, and Mig1p and Mig2p (Wang *et al*. 2017) are two homologues of glucose repressor Mig1p of *S. cerevisiae*. However, how these transcription regulators interact with each other to regulate MUT genes has not been elucidated.

The expression of genes encoding transcription factors is also regulated by transcription factors, unless it is constitutive. It can be self-regulated (Sasano *et al*. 2008; Wang *et al*. 2016b) or controlled by another transcription factor (Wang *et al*. 2016b). It is indicated that engineering the promoter region of transcription factors could alter the induction condition of the transcription factor followed by altering the expression of entire genes under the regulation of the transcription factor. This was demonstrated previously using KpTrm1p, formerly Prm1p, at the first time (Takagi *et al*. 2012).

A couple of methanol-independent expression systems based on the *AOX1* promoter were successfully established in *K. phaffii* by altering the promoter of positive regulators, namely, Mit1p and Mxr1p. Their promoters were replaced with a *CAT* promoter (Vogl *et al*. 2018) or an *AOX2* promoter (Chang *et al*. 2018); both work under carbon depleted (de-repressed) conditions without methanol. Both systems outperformed standard production with methanol >2-fold on model proteins. Replacing the promoter of *MIT1* with a constitutive *GAP* promoter and deletion of glucose repressors Mig1p/Mig2p/Nrg1p were combined with a carbon source shifting fermentation from glucose to glycerol to induce the *AOX1* promoter (Wang *et al*. 2017). Also, it was discovered that deleting two kinases—Δ*gut1* or Δ*dak*—induces the *AOX1* promoter with nonmethanol carbon sources (Shen *et al*. 2016). However, these systems reached protein productivity at 50–60% of methanol induction.

There are a couple of examples showing that the multiplication of an Upstream Activation Sequence (UAS) containing the *cis*-element of a positive transcription factor enhanced the induction of the promoter (Ohi *et al*. 1994; Minetoki *et al*. 1998). Ohi *et al*. (1994) reported that multiplying the UAS of the *AOX2* promoter by three copies increased the production of human serum albumin (HAS) in *K. phaffii* over 50 times. In a fungus *Aspergillus oryzae*, 12 tandem repeats of region III, which is a conserved region among amylase genes in *A. oryzae*, increased the expression of the *agdA* promoter >20-fold (Minetoki *et al*. 1998).

Here, we report on our attempt to develop a methanol-free expression system of *K. phaffii*, using another strong methanol-inducible *DAS1* promoter through engineering its transcription factors, namely KpTrm1p, which was applied to the production of an industrial enzyme in high-cell-density fermentation. Multiplication of the UAS of the *DAS1* promoter was also tested in order to increase enzyme productivity with and without methanol. We also discuss our findings on the difference in the regulation of the *AOX1* promoter and the *DAS1* promoter by known transcription factors in *K. phaffii*.

## MATERIALS AND METHODS

### Strains, vectors and media

*K. phaffii (Pichia pastoris)* GS115 (*his4*, Mut^+^), KM71 (*∆aox1, his4*, Mut^S^) and expression vectors pPIC9K, pGAPZαA were purchased from Invitrogen (Life Technologies, Tokyo, Japan). *Escherichia coli* cloning vector pCR2.1-TOPO (Life Technologies, Tokyo, Japan) and pT7Blue-Novagen (Sigma-Aldrich Japan, Tokyo, Japan) and cloning host *E. coli* DH5α (TOYOBO Co. Ltd, Osaka, Japan), TOP10 (Life Technologies), XL10 (Stratagene, CA, USA) were purchased from suppliers. Yeast strains were grown on YPD (1% yeast extract, 2% peptone, 2% dextrose) unless specified. Media used for phytase production were described below. *E. coli* strains were grown in LB (1.0% Trypton, 0.5% yeast extract, 0.5% NaCl) with relevant antibiotics. Other strains and plasmids used in this study are summarized in Tables S1 and S2 (Supporting Information). Mut^+^ and Mut^S^ represent methanol-utilization plus and methanol-utilization slow phenotype, respectively.

### Expression plasmids

A codon-optimized synthetic gene encoding *Citrobacter braakii* phytase G01651 and its expression plasmid with *AOX1* promoter, pPICNoT-G01651, was described in US 8.236,528 B2 (Takagi *et al*. 2012). The same synthetic gene was subcloned into pGAPZαA, generating pGAPα-G01651, to express phytase with *GAP* promoter. An orthologue of *C. boidinii DAS1* gene (Genbank: AF086822) encoding dihydroxyacetone synthase was identified in the *K. phaffii* genome sequence provided by Integrated Genomics, Inc. (current Igenbio, Inc. Chicago, IL, US) (Figure S1, Supporting Information). About 1 kb of 5'-untranslated region of *DAS1* gene (GenBank GZ456654) was isolated from *K. phaffii* GS115 and used for construction of phytase expression plasmid pNo-DP3 (Takagi *et al*. 2012). Construction of *DAS1* promoter variants and their phytase expression plasmids were described in WO 2010/0 04042 (Tsutsumi and Takagi 2010). These plasmids were integrated at *HIS4* locus of the host strain as a single copy to compare each other. Cloning of *K. phaffii* GS115 orthologue of *TRM1* from *C. boidinii* (GenBank AB365355), termed *KpTRM1*, formerly called *PRM1* (GenBank GZ456640), and another positive regulation factor *MXR1* (Lin-Cereghino *et al*. 2006), and their constitutive expression plasmids, pGPrm and pGMxr, using *GAP* promoter were described in US 8.236,528 B2 (Takagi *et al*. 2012).

### Transformation of *K. phaffii*

*K. phaffii* strains were transformed by electroporation following the manufacturer's protocol (Invitrogen, Cat. No. K1710–01). RD medium (1 M sorbitol, 2% dextrose, 1.34% yeast nitrogen base (YNB), 4 × 10^−5^% biotin, 0.005% each amino acid of L-glutamic acid, L-methionine, L-lysine, L-leucine, L-isoleucine) supplemented with 2% Difco Agar Noble or MD medium (2% dextrose, 1.34% YNB, 4 × 10^−5^% biotin) supplemented with 1 M sorbitol and 2% agar noble was used for regeneration with *his4* selection. In the case of Zeocin resistance selection, YPDS + Zeocin agar (1 M sorbitol, 2% dextrose, 1% yeast extract, 2% peptone, 2% bacto agar, 100 μg/mL Zeocin (Life Technologies, Tokyo, Japan)) was used for the regeneration. Plates were incubated at 28 or 30°C for 3–4 days until colonies appeared. Screening for Mut^+^/Mut^S^ phenotypes was performed using MD (minimum dextrose) medium plates and MM (minimum methanol) medium plates following the published protocol (Invitrogen Catalog Number K1710–01).

### Colony PCR for screening for integration at the *HIS4* locus

A small portion of a colony grown on agar medium was picked with a sterile toothpick and transferred into a small tube, then heated in a microwave oven for one minute. The dried cells were suspended in 50 μL sterilized water and subjected to PCR. The reaction mixture was 20 μL including 2 mM dNTP, 10 μM of each primer, 1 unit of Expand high fidelity plus (Roche Diagnostics K.K., Tokyo, Japan), 1 × Expand high fidelity plus buffer and 1 μL of cell suspension as mentioned above. The PCR primers were primer H1: 5’-CTGCTCTAGCCAGTTTGCTG -3’ corresponding the sequence upstream of *HIS4* in the host genome, and primer H2: 5’-GCCGCCCAGTCCTGCTCGCT-3’based on the sequence in the expression plasmids. The strains in which the expression cassette was integrated at *HIS4* locus generated a 2.9 kb band.

### Phytase expression test

Isolated transformants were grown in liquid medium to evaluate the phytase expression level. In the case of methanol induction, strains were grown in YPD with vigorous shaking at 30°C for 2 days, then methanol was added at the final concentration 0.5–2% (V/V) to induce the expression. Activity measurement was done using 3 days’ sample. In the case of expression without methanol, strains were grown in YPD with vigorous shaking. Glucose in YPD medium was usually consumed within 2–3 days in the tested condition. Two days’ or 3 days’ samples were used for activity measurement. Phytase activity was measured as described before (Takagi *et al*. 2012).

### High-cell-density fermentation in lab fermenter

High-cell-density fermentation was performed following the manufacturer's protocol (*Pichia* Fermentation Process Guidelines, version B 05 3002, 2002 Invitrogen Corporation) with some modification. Fermentation was conducted in 5 L-fermenters (ABLE Corporation, Tokyo, Japan) starting with 2 liters of basal medium (85% H_3_PO_4_ 26.7 mL/L, CaSO_4._2H_2_O 1.1 g/L, K_2_SO_4_ 18.2 g/L, MgSO_4_.7H_2_O 14.9 g/L, KOH 4.1 g/L, glycerol 40.0 g/L). Strains were pre-cultured in 110 mL of YPD at 30°C for 1 day, then transferred to the basal medium supplemented with PTM_1_ Trace Salts (Invitrogen). The fermentation was controlled at 30°C, pH above 5.0 and keeping dissolved oxygen (DO) level over 30% by adjusting agitation speed. Glycerol feeding started at 12 hours and the feeding rate was increased step-by-step until 40 hours. When the DO level decreased below 10%, feeding was stopped until the DO recovered. In the case of the methanol fermentation, methanol feeding was started at 40 hours. The methanol-feeding rate after 41 hours was adjusted aiming to maintain the methanol concentration in the medium at 0.8%. When the DO level decreased below 20%, methanol feeding was stopped until the DO recovered and then the feeding rate was decreased to keep DO level over 20%. In the case of glucose fermentation, initial carbon source in the basal medium and feeding substrate was glucose instead of glycerol. Glucose feeding started at 12 hours and feeding rate was increased step-by-step until 40 hours. After 40 hours, the feeding rate was controlled to keep the DO level between 10–30%. Residual glucose in the medium was usually below 0.2 g/L after ∼70 hours. Dried cell weight (DCW) was measured as follows; ∼2 mL of culture broth was transferred to a pre-weighed glass tube and the tube was again weighed to calculate a weight of the whole broth. Cells were collected from the broth by centrifugation at 3500 rpm for 10 minutes, then washed twice with Reverse Osmosis (RO) water. After discarding the washed water, cells in the tube were dried in drying oven at 105°C for at least 24 hours. After cooling in a desiccator, the tube was weighed to calculate the weight of dried cells. Dried cell weight (DCW/g) was obtained as a quotient of the weight of dried cells divided by the weight of whole broth. Phytase activity was measured using the culture supernatant after centrifugation.

## RESULTS AND DISCUSSION

### Expression of bacterial phytase in *K. phaffii*

A codon optimized synthetic gene of *C. braakii* phytase was tested in *K. phaffii* KM71 using the *AOX1* promoter for its expression to compare with the native phytase gene. Phytase production was evaluated in high-cell-density fermentations with methanol feeding. As shown in Table 1; strain AOX36—with the codon-optimized synthetic gene—produced double the amount of phytase compared with strain AOX3, which carries the native gene. A higher phytase yield was achieved by strain AOX94 which was generated with another host strain, *K. phaffii* GS115, although the reason for this higher yield was not known.

**Table 1.** Phytase production with *K. phaffii* in high-cell-density fermentations.

Strain code	Mut phenotype	Host strain	Promoter for phytase	Gene type of phytase	Data at 168 hours		
					Phytase yield (unit/mL)	DCW (mg/g)	DCW (unit/mg)
Methanol fermentation							
AOX3	Mut^S^	KM71	*AOX1*	Native	1100	90	12.2
AOX36	Mut^S^	KM71	*AOX1*	Synthetic	2200	90	24.4
AOX94	Mut^S^	GS115	*AOX1*	Synthetic	4000	117	34.1
DAS40	Mut^+^	GS115	*DAS1*	Synthetic	5140	110	46.7
28-2	Mut^+^	GS115	*DAS1^t^ x 3*	Synthetic	7290	134	54.4
Glucose fermentation							
GAP46H^a^	Mut^+^	GS115	*GAP*	Synthetic	4000	180	22.2
2P-4	Mut^+^	2–3	*DAS1^t^*	Synthetic	4960	148	33.5

a The original transformant isolated with Zeocin selection, GAP46, had a *his*- phenotype, therefore, it was transformed with an empty expression vector, pPIC9K, to complement *HIS4* resulting in GAP46H.

*DAS1^t^*: truncated version of *DAS1* promoter, *DAS1^t^ x 3*: *DAS1^t^* promoter with three copies of UAS_DAS1_.

**Figure 1.**
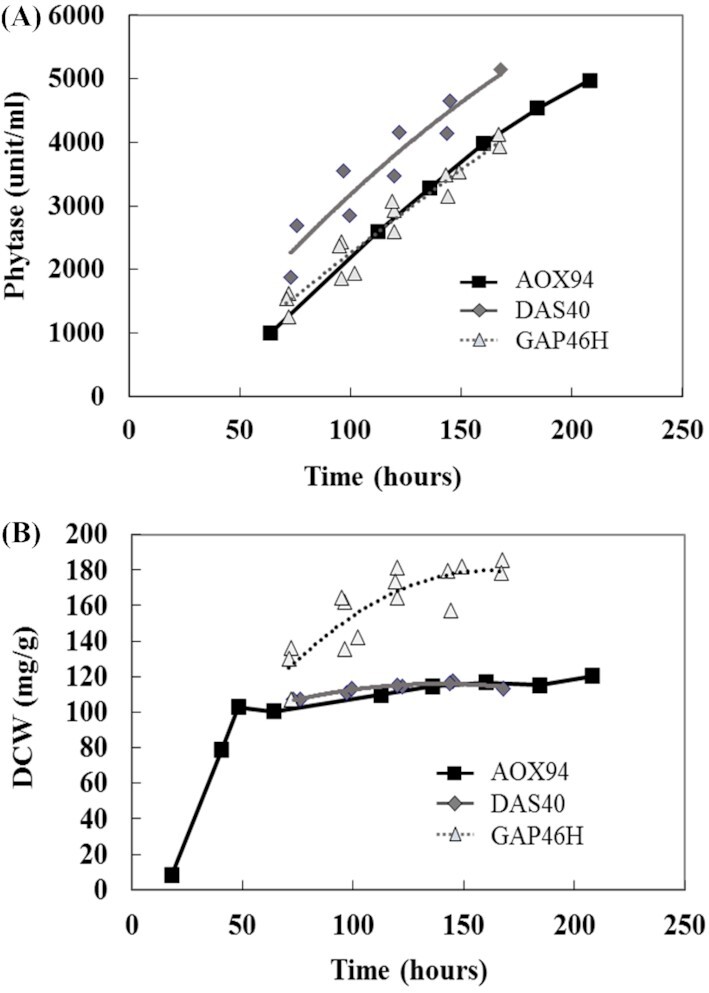
Comparison of phytase expression using different promoters in high-cell-density fermentations. **(A)** Phytase activity in the culture supernatant (unit/ml). **(B)** Dried cell weight (DCW) per culture broth (mg/g). Closed squares: AOX94 with the *AOX1* promoter, closed diamonds: DAS40 with the *DAS1* promoter (*n* = 2), gray triangles: GAP46H with the *GAP* promoter (*n* = 3). AOX94 and DAS40 were fermented with methanol feeding, GAP46H was fermented with glucose feeding. The genotype of each strain is shown in Table S1 (Supporting Information).

Strains producing phytase constitutively were generated from *K. phaffii* GS115 using the pGAPα-G01651 expressing phytase with the *GAP* promoter. A selected strain, GAP46H, was tested in high-cell density fermentation with glucose feeding. In the case of methanol fermentation, strain AOX94 grew poorly in the methanol feeding phase due to its Mut^S^ (methanol utilization slow) phenotype and maintained the cell mass during the enzyme production phase (Fig. 1). On the other hand, in the case of glucose fermentation, the strain GAP46H continuously increased the cell mass during the glucose-feeding phase, and the phytase production yield increased along with the cell mass. Eventually, after 168 hours, the phytase production yield with the *GAP* promoter reached a level comparable with the yield using the *AOX1* promoter (Fig. 1). However, enzyme productivity per dried cell mass was 35% lower with strain GAP46H with the *GAP* promoter than with strain AOX94 with the *AOX1* promoter.

The *DAS1* promoter, which is most strongly induced by methanol in *C. boidinii* (Yurimoto *et al*. 2000), was also tested for phytase production in *K. phaffii*. A selected transformant, DAS40, was tested in high-cell density fermentation with methanol feeding in the same manner as the strain AOX94 with the *AOX1* promoter (Fig. 1). In the case of DAS40, an attempt was made to insert the plasmid at *Sna*BI site in the *DAS1* promoter region by single cross-over, which should not affect the expression of the endogenous *DAS1* gene, keeping the Mut^+^ (Methanol utilization plus) phenotype of the strain. However, the cell mass of strain DAS40 did not increase during the methanol feeding phase (Fig. 1B). This could be because some DNA rearrangement occurred during the transformation event, affecting the Mut^+^ phenotype, but this needs to be studied more in order to be conclusive. Strain DAS40 with the *DAS1* promoter reached a phytase yield of around 30% higher than the yield of strain AOX94 with the *AOX1* promoter.

### Methanol-free expression system by engineering of *KpTRM1*

**Figure 2.**
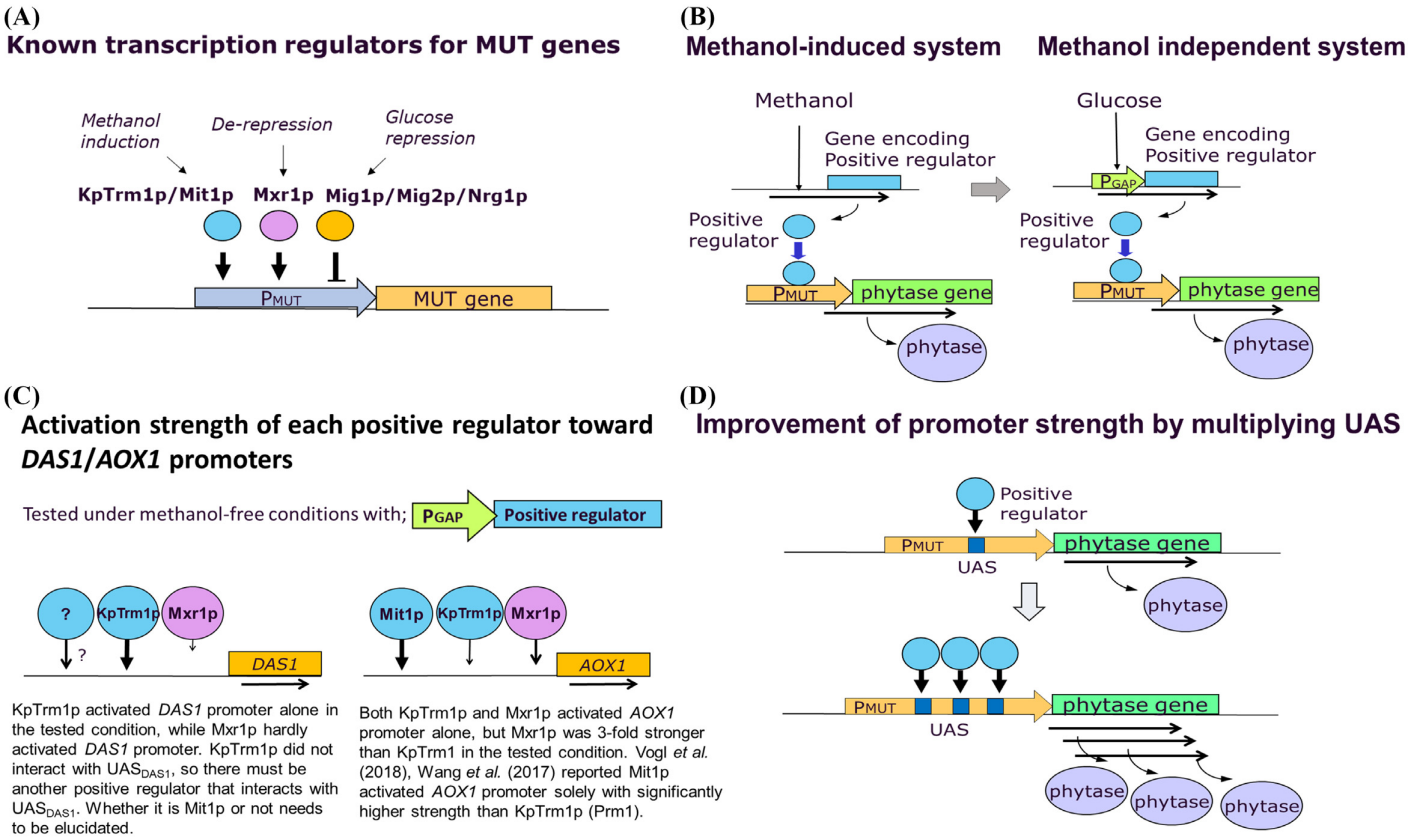
Illustration of background idea of developed technology and summary of regulation of *DAS1/AOX1* promoters. **(A)** There are three types of regulators known for MUT genes in *K. phaffii*; 1) positive regulator under methanol induction, KpTrm1p (formerly Prm1p), Mit1p, 2) positive regulator under de-repressed conditions, Mxr1p, 3) negative regulator under glucose repression, Mig1p, Mig2p, Nrg1p. These regulators interact with the specific regions of MUT promoters depending on the surrounding conditions. **(B)** illustrates the idea of conversion of methanol inducible expression system to methanol-free expression system. By replacing the promoter of the gene encoding a positive regulator of MUT genes with a constitutive *GAP* promoter, MUT promoter will be activated constitutively. **(C)** illustrates the activation strength of each regulator to the *DAS1* promoter and the *AOX1* promoter based on the results of this work and learning from the literature. Activity strength is shown by thickness and length of arrow. **(D)** UAS is Upstream Activation Sequence which a positive regulator interacts with to activate promoter. When multiplying UAS, promoter activity could be increased accordingly.

**Figure 3.**
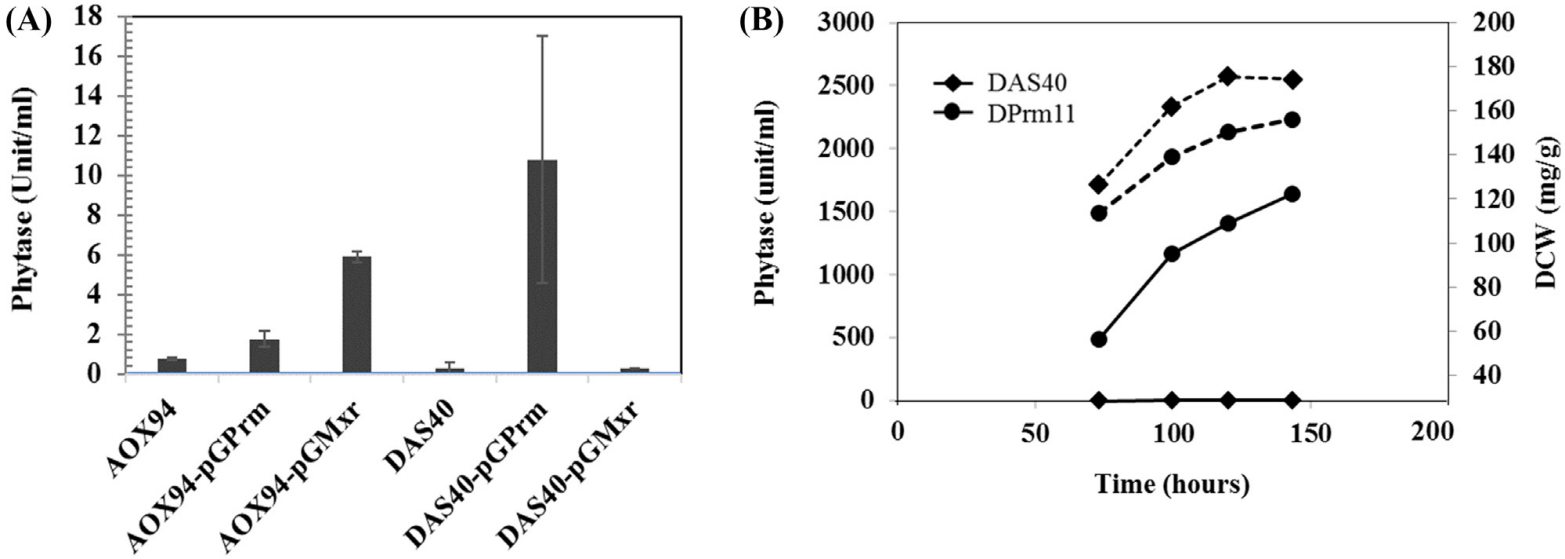
Effect of constitutively expressed regulators on methanol-free expression. **(A)** Phytase expression with glucose medium in shaking flasks. AOX94 and DAS40 are the reference strains expressing phytase under the *AOX1* promoter or the *DAS1* promoter, respectively. KpTrm1p (pGPrm) and Mxr1p (pGMxr) were constitutively co-expressed in AOX94 or DAS40. Average yields of multiple isolates from each combination are shown. **(B)** High-cell-density fermentations under methanol-free condition (glucose feeding). DAS40 (closed diamond) is a strain expressing phytase under the *DAS1* promoter and DPrm11 (closed circle) is a strain derived from DAS40 by co-expressing constitutive KpTrm1p (pGPrm). Solid lines show phytase activities (unit/mL), broken lines show dried cell weights (DCW). DAS40 grew well in the methanol-free condition, but did not produce phytase.

Several positive transcription factors for MUT genes in methylotrophic yeast have been reported (Yurimoto, Oku and Sakai 2011; Vogl *et al*. 2018) (Fig. 2A). Trm2p of *C. boidinii* (Sasano *et al*. 2010) and Mxr1p of *K. phaffii* (*P. pastoris*) (Lin-Cereghino *et al*. 2006) are homologues of *S. cerevisiae* Adr1p which is known as a positive regulator working under de-repressed conditions. Trm1p of *C. boidinii* (Sasano *et al*. 2008) and Mit1p of *K. phaffii (P. pastoris)* (Wang *et al*. 2016b) are Gal4 type transcription factors that are likely true positive regulators of the methanol induction of MUT genes. Among them, Trm1p was reported as a master regulator of methanol-specific induction in *C. boidinii* (Sasano *et al*. 2008), so we selected it as the first target of engineering when we attempt to construct a methanol-free expression system. An orthologue of *TRM1* in *K. phaffii*, *KpTRM1—*formerly called *PRM1*—was isolated from the genome of *K. phaffii* GS115 and confirmed to be an essential factor for growth with methanol (Figure S2, Supporting Information). We expected that the expression driven by the promoter of the MUT gene could be controllable via the engineering of this transcription factor (Fig. 2B) and the constitutive expression plasmid pGPrm was constructed (Takagi *et al*. 2012). It was introduced into the above-mentioned strains, AOX94 and DAS40, and tested for methanol-free phytase expression. Phytase productivity was evaluated by cultivating cells in shaking flasks with YPD medium for 2 to 3 days without adding methanol. Under the tested conditions, glucose in the medium was used up within 2 to 3 days, which means that the late stage of cultivation was under glucose depleted condition. As the results show, reference strains AOX94 and DAS40 produced very little phytase, while the derived strains co-expressing constitutive *KpTRM1* with pGPrm showed significantly higher phytase activity (Fig. 3A). However, compared with the expression yield with methanol induction, the observed phytase yield was over 50 times less in the case of the *AOX1* promoter (data not shown). Surprisingly, the effect of the pGPrm on the *DAS1* promoter was much more significant than its effect on the *AOX1* promoter. Selected co-expressing strain DPrm11, producing phytase with the *DAS1* promoter, was evaluated in high-cell-density fermentation with glucose feeding in order to compare it with reference strain DAS40 (Fig. 3B). The glucose feeding rate was controlled to maintain the residual glucose level below 0.2 g/L during enzyme production, in order to avoid glucose repression. Strain DPrm11, co-expressing constitutive *KpTRM1*, produced significant amount of phytase, while parent strain DAS40 did not show phytase activity, although the strain grew well (Fig. 3B). The phytase yield of DPrm11 was ∼40% of the yield obtained by GAP46H with *GAP* promoter.

### Difference between *MXR1* and *KpTRM1*’s effects on methanol-inducible promoters

Another positive regulator of a methanol-inducible promoter, Mxr1p (Lin-Cereghino *et al*. 2006), was also examined. Plasmid pGMxr expressing *MXR1* constitutively with a GAP promoter was introduced into the same reference strains, AOX94 and DAS40, and tested in a similar manner. Interestingly, unlike KpTrm1p, the effect of Mxr1p on the *DAS1* promoter was negligible, while its effect on the *AOX1* promoter was more significant than the effect of KpTrm1p (Fig. 3A). Considering that Mxr1p is a presumed positive regular working under de-repressed conditions, the obtained results were consistent with findings in *C. boidinii* (Yurimoto and Sakai 2009), which showed that the *AOD1* (*AOX1* homologue) promoter was partially activated under de-repressed conditions without methanol feeding, while the *DAS1* promoter was not activated under de-repressed conditions and required the addition of methanol. Yurimoto, Oku and Sakai (2011) indicated that the induction of the *DAS1* promoter was mainly achieved with methanol-specific induction by Trm1p in *C. boidinii*, although the de-repression by Trm2p was necessary for the induction of this promoter. Thus, the contribution of Trm2p (Mxr1p homologue) to the methanol-specific induction of the *DAS1* promoter was smaller than that of Trm1p in *C. boidinii*. Our results suggest that it is probably the same in *K. phaffii*, i.e. the *DAS1* promoter is mainly activated by methanol-induction specific KpTrm1p, and Mxr1p alone does not induce the *DAS1* promoter (Fig. 2C). Whether Mxr1p was necessary for KpTrm1p to activate the *DAS1* promoter was not conclusively established, because the tested fermentation conditions provided glucose-depleted conditions in the late stage of fermentation, which could activate endogenous Mxr1p. This remains to be confirmed by further research.

**Figure 4.**
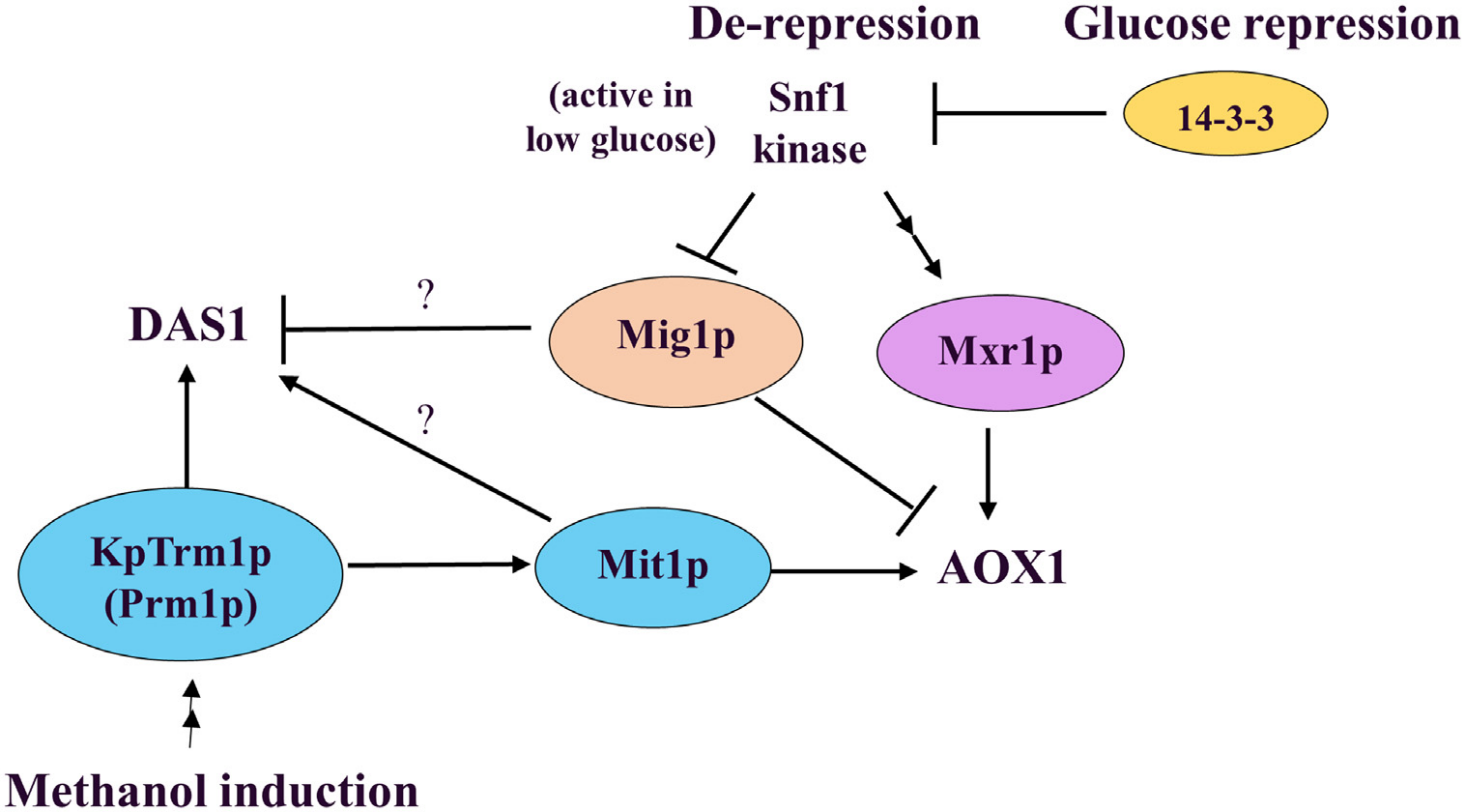
Presumed regulation of the *AOX1* and the *DAS1* by known transcription regulators in *K. phaffii*. *AOX1* requires Mit1p to be active with methanol through KpTrm1p (Prm1p), while *DAS1* can be solely activated by KpTrm1p. *AOX1* is also activated by Mxr1p in the glucose-depleted conditions via, e.g. Snf1 kinase, while *DAS1* is likely independent to Mxr1p. See text for more information.

In the case of the *AOX1* promoter, co-expression of constitutive Mxr1p was three times more effective than KpTrm1p in a methanol-free condition (Fig. 3A). However, the yield was still >10 times less than the yield with methanol induction (data not shown), which indicates that another important regulator exists for the *AOX1* promoter. Wang (2017) and Vogl *et al*. (2018) reported that overexpression of Mit1p, another positive regulator of the Gal4 type, was more effective than KpTrm1p (Prm1p) on the methanol-independent expression of *AOX1* in *K. phaffii* (*P. pastoris*). Their results indicate that Mit1p is the true methanol-induction-specific regulator for *AOX1*, rather than KpTrm1p. Wang (2016b) reported that KpTrm1p (Prm1p) binds the promoter of *MIT1* to activate its expression in the presence of methanol and suggested that KpTrm1 could be a receptor of the methanol signal and transfers it to *MIT1*. The results showed that KpTrm1p was still an important regulator for the *AOX1* promoter under methanol induction. This suggests that the regulation of the *AOX1* promoter is more complex and involves multiple regulators, while the regulation of the *DAS1* promoter is simpler and involves fewer regulators. Our work did not produce new findings about the effect of glucose repression factors Mig1p/Mig2p/Nrg1p (Fig. 2A) on the *DAS1* promoter; however, it is known that Mig1p in *S. cerevisiae* is inactivated by Snf1 kinase in glucose-depleted conditions (Klein, Olsson and Nielsen 1998). At the same time, Snf1 likely activates Adr1 under the same conditions (Turcotte *et al*. 2010). Similar regulation can be expected on glucose repressors such as Mig1p and the Adr1p homologue Mxr1p in *K. phaffii* (*P. pastoris*). Li *et al*. (2018) also indicated that the AMPK/SNF1 pathway is likely related to the activation of the *AOX1* promoter in *K. phaffii* via Mxr1p, Mig1p, and 14–3-3 (Parua *et al*. 2012). The presumed regulation on *AOX1* and *DAS1* by known regulation factors is illustrated in Fig. 4.

### UAS_DAS1_ for the methanol induction of the *DAS1* promoter

Aiming to engineer a methanol-inducible promoter to enhance promoter activity (Fig. 2D), we attempted to identify an UAS in the *DAS1* promoter (Tsutsumi and Takagi 2010). Deletion variants of the *DAS1* promoter were constructed and evaluated for phytase production in shaking flasks with methanol, as shown in Figure S3A (Supporting Information). Interestingly, pDd-2—which had 200-bases of deletion between −1055 and −855—increased phytase productivity by 50% compared with the pNo-DP3 carrying the original *DAS1* promoter of 1-kb. Similarly, pDd-4, in which the region between −755 and −655 was deleted, increased phytase yield by 28% compared with pDd-3. Results indicated that these regions contained an upstream repression sequence (URS) and deletion of the URS increased phytase expression. Ohi *et al*. (1994) reported a similar finding, namely that two URSs existed in the *AOX2* promoter of *K. phaffii* (*P. pastoris*), at around −645 and −255, and that removing or mutating these URSs increased the expression level dramatically.

Further deletion work successfully identified the region at −355 to −255 as essential to the methanol induction of the *DAS1* promoter (Figure S3B, Supporting Information). The region was named UAS1_DAS1_. Sasano et al. (2008) reported that two methanol response elements—MRE1 (*CCTATTCCAAAAAGGG*) and MRE2 (*TGCATTCCTAAAATAG*)—existed in the *DAS1* promoter of *C. boidinii* and that MRE1 was closely related to the induction by Trm1p. Similar sequences were identified in the UAS1_DAS1_ in *K. phaffii*, termed PBS1 and PBS2, and their deletion was examined (Tsutsumi and Takagi 2010). Deletion of PBS1 or PBS2 reduced the expression level by 25% and 75%, respectively. The impact of the deletion of each region, however, was smaller than the impact of the deletion of entire UAS1_DAS1_. It was decided to use the whole UAS_DAS1_ for further engineering work.

### Engineering of the *DAS1* promoter: amplification of UAS1_DAS1_

**Figure 5.**
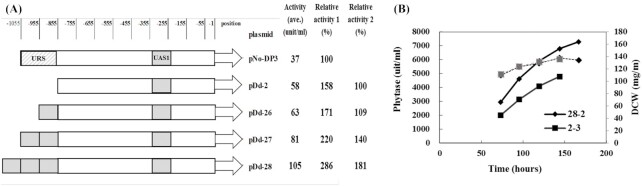
Effect of amplification of UAS1_DAS1_ in the *DAS1* promoter. Expressions were tested in the presence of methanol. **(A)** Structures of *DAS1* promoter variants with amplified UAS1_DAS1_ and phytase yields in shaking flasks. Phytase yield increased along with the amplification of UAS1_DAS1_. **(B)** Phytase production in high-cell-density fermentation under methanol feeding. Strain 28-2 (closed diamond) carried pDd-28 with three extra UAS1_DAS1._ Strain 2-3 (closed squares) was a reference strain generated with the pDd-2. Solid lines show phytase activity (unit/ml) and broken lines show dried cell weight (DCW) (mg/g).

The amplification of UAS1_DAS1_ was tested using the truncated version of the *DAS1* promoter, without URS. UAS1_DAS1_ was amplified up to three copies in tandem and fused to the 5’-upstream of the *DAS1* promoter on pDd-2 (Fig. 5A). Plasmids generated with this construction were integrated at the *HIS4* locus of host strain GS115 in single copy and tested for phytase production with methanol in a shaking flask test. Phytase activity increased along with the amplification of UAS1_DAS1_. The variant pDd-28, with three extra UAS1_DAS1_, increased phytase production by 80% compared to pDd-2. It was over twofold higher than the reference with pNo-DP3. Selected strain 28-2, generated with pDd-28, was evaluated in high-cell-density fermentations with methanol induction and compared with strain 2-3 carrying pDd-2. The phytase yield of 28-2 was 40% higher than that of strain 2-3 (Fig. 5B). It was ∼1.4-fold of the yield by DAS40 carrying pNo-DP3 with the original 1 kb *DAS1* promoter, and 1.8-fold compared with the yield of AOX94 with the *AOX1* promoter. It was the highest phytase yield with methanol that was achieved during the research.

### Methanol-free expression with an engineered *DAS1* promoter

Co-expression of constitutive *KpTRM1* was tested for phytase expression with *DAS1* promoter variants. The plasmid pGPrm was introduced into phytase-producing strains generated with the plasmids pDd-14∼pDd-20, and methanol-free enzyme production was studied in shaking flasks (Table 2). As expected, none of the strains without co-expression of pGPrm produced phytase with glucose; therefore, the observed phytase activities resulted from constitutively-expressed KpTrm1p. Interestingly, strains generated with the plasmid in which UAS1_DAS1_ had been deleted (pDd-14 and pDd-20) showed higher phytase activity than the plasmids maintaining UAS1_DAS1_ (pPd-17∼19). The results suggest that UAS1_DAS1_ was not a *cis*-element of KpTrm1p for activating the *DAS1* promoter in the tested methanol-free expression system. This unexpected result indicated that there must be another element in the *DAS1* promoter for KpTrm1p to interact with. Careful analysis revealed that the region between −455 and −355 was more important to maximizing the effect of KpTrm1p (pDd-14, 19 and 20, Table 2). The region was named ESP_DAS1_, enhancing sequence for KpTrm1p (Prm1p). In fact, strains carrying these plasmids, except 19P-13 with pDd-19, showed similar levels of phytase productivity to DPrm11 carrying the wild type *DAS1* promoter. The reason for the lower yield of 19P-13 is not known, but it might be related to the region deleted in plasmid pDd-19. Moreover, there must be another regulation factor that interacts with UAS1_DAS1_ to enhance methanol activity_._ Determining whether it is Mit1p requires further study.

**Table 2.** Methanol-free phytase production using *DAS1* promoter variants with or without co-expression of constitutive *KpTrm1*.

Strain	Host	Plasmid for phytase expression			pGPrm co-expression	Phytase activity/cell mass (units/OD660)
		Plasmid for phytase expression	UAS1_DAS1_^a^ (−355∼−255)	ESP_DAS1_^a^ (−455∼−355)		
14-1	GS115	pDd-14	−	+	no	0
16-3	GS115	pDd-16	−	−	no	0
17-5	GS115	pDd-17	+	−	no	0
18-3	GS115	pDd-18	+	−	no	0
19-2	GS115	pDd-19	+	+	no	0
20-1	GS115	pDd-20	−	+	no	0
14P-5	14-1	pDd-14	−	+	yes	9.46
16P-7	16-3	pDd-16	−	−	yes	0.68
17P-9	17-5	pDd-17	+	−	yes	1.48
18P-12	18-3	pDd-18	+	−	yes	1.99
19P-13	19-2	pDd-19	+	+	yes	2.79
20P-15	20-1	pDd-20	−	+	yes	8.28
DPrm11	DAS40	pNo-DP3	+	+	yes	9.89

a UAS_DAS1_ is an essential region for the methanol induction of the *DAS1* promoter. ESP_DAS1_ is the region which enhances the effect of constitutive *KpTrm1* (pGPrm) on a methanol-free expression using the *DAS1* promoter.

**Figure 6.**
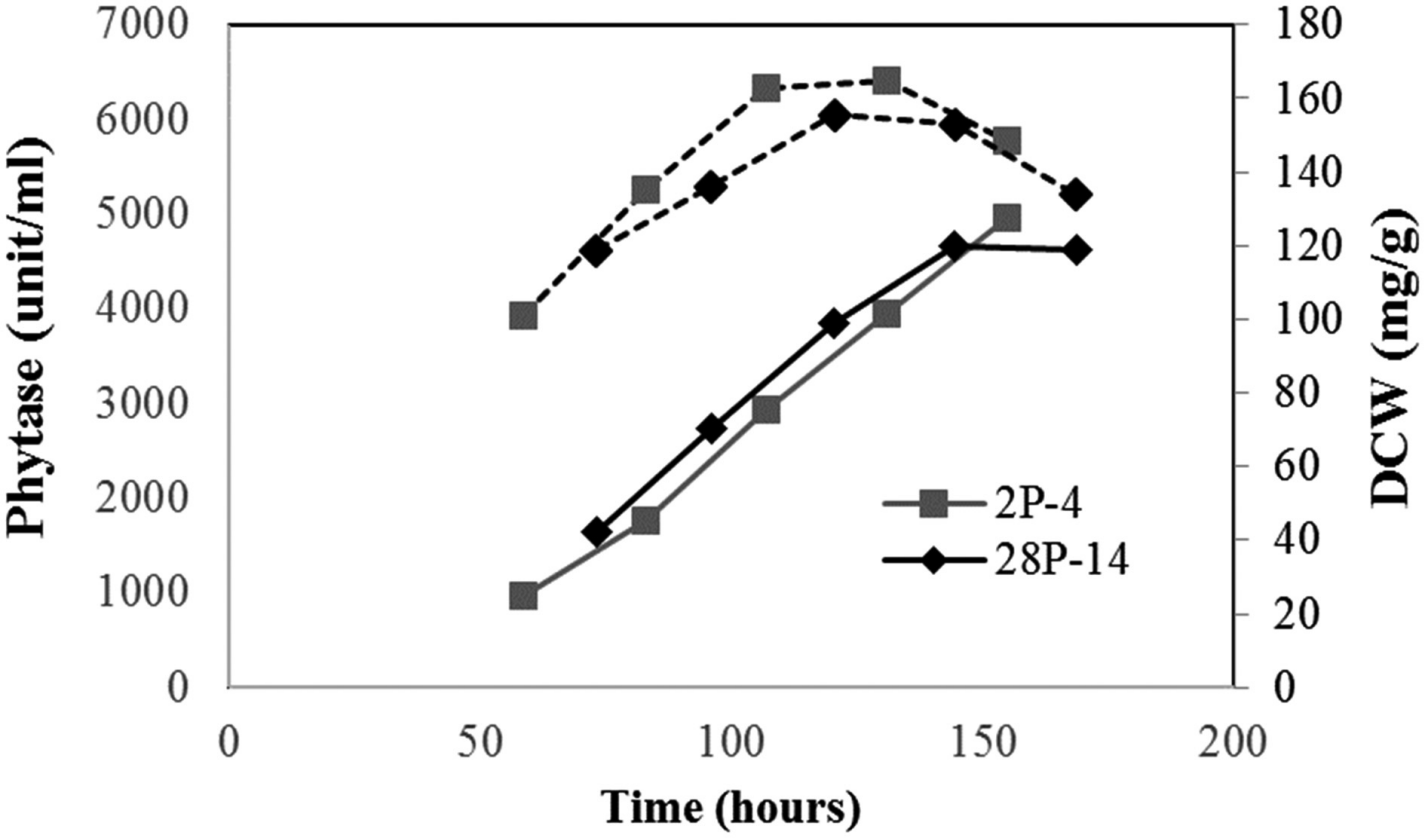
Methanol-free phytase expression using the *DAS1* promoter variants in high-cell-density fermentations with glucose feeding. 2P-4 (closed diamonds) and 28P-14 (closed squares) are strains derived from 2-3 or 28-2 by co-expressing constitutive KpTrm1p (pGPrm). Phytase yields (unit/mL) are shown in solid lines. Dried cell weights (mg/g) are shown in broken lines.

Constitutive KpTrm1p was also tested in strain 28-2 carrying the *DAS1* promoter, with 3xUAS1_DAS1_ for phytase production. Selected strain 28P-14, derived from strain 28-2, and strain 2P-4, derived from reference strain 2-3, were evaluated in high-cell-density fermentations under methanol-free conditions (Fig. 6). As expected, there was no significant difference between phytase expressions with these two strains, confirming that the UAS1_DAS1_ does not interact with KpTrm1p in the tested methanol-free fermentation. Interestingly, the phytase yields obtained with these two strains were three times higher than the previously constructed DPrm11 derived from strain DAS40, carrying pNo-DP3 with the original *DAS1* promoter of 1-kb for phytase expression. This might be because the truncated version of the *DAS1* promoter was used for the construction of pDd-2 and pDd-28. One possible reason could be that the removed −855∼−1055 region contained URS—which affects the promoter activity negatively, even without methanol. Glucose repression could be an example of such a negative effect, which would have a significant impact, especially in glucose fermentation. Ohi *et al*. (1994), however, reported that neither of the identified URSs, URS1 (−645∼−684) and URS2 (−255∼−215), in the *AOX2* promoter were involved in catabolite repression, and the mechanism of their repressing effect was not defined. Another possible reason for the yield increase could be the integration locus of the expression plasmid. In the case of 2-3 and 28-2, plasmids pDd-2 and pDd-28 were integrated at the *HIS4* locus, while in the case of DAS40, pNo-DP3 was integrated at the *DAS1* promoter region. To elucidate mechanisms of improvement, further experiments are awaited. Nevertheless, the newly developed methanol-free expression system based on a truncated version of the *DAS1* promoter produced 25% more phytase than the system based on the *GAP* promoter (Fig. 6). It was the highest yield of phytase achieved in methanol-free conditions. Further evaluation of ESP_DAS1_ on the effect of a methanol-free system is awaited.

## SUMMARY

Results obtained during our research revealed that transcriptional regulation between two MUT genes, *AOX1* and *DAS1*, in *K. phaffii* was different (Fig. 4). In the case of *DAS1*, single transcription factor KpTrm1p was enough to activate the *DAS1* promoter, while *AOX1* required another transcription factor, such as Mit1p or Mxr1p, to fully activate its promoter. This difference made it possible to develop a methanol-free expression system using the *DAS1* promoter and KpTrmp1, but not the *AOX1* promoter.

Multiplying UAS_DAS1_ and removing URS in the *DAS1* promoter enhanced methanol induction of the *DAS1* promoter significantly. However, it turned out that UAS_DAS1_ was not a *cis*-element of KpTrm1p, and its amplification did not affect methanol-free expression by constitutive KpTrmp1. Another region termed ESP_DAS1_ was identified instead as a potential *cis*-element of KpTrm1p. Nevertheless, the engineered *DAS1* promoter combined with constitutive KpTrm1p successfully surpassed phytase production with the *GAP* promoter.

## Supplementary Material

foz059_Supplemental_File
