# Supplementary material for: Engineering the expression system for Komagataella phaffii (Pichia pastoris): an attempt to develop a methanol-free expression system
Source: FEMS Yeast Res. 2019 Aug 13;19(6):foz059. doi: 10.1093/femsyr/foz059 (PMC6736287; doi:10.1093/femsyr/foz059)
Supplement: foz059_Supplemental_File [file foz059_supplemental_file.docx]

Supplemental data

**Engineering the expression system for *Komagataella phaffii (Pichia pastoris)*: an attempt to develop a methanol-free expression system**

Shinobu Takagi^1§*^, Noriko Tsutsumi^1§^, Yuji Terui^1^, XiangYu Kong^2^, Zheng Liu^2^, Hiroya Yurimoto^3^, Yasuyoshi Sakai^3^ (^§^Equally contributed, *Correspondence)

1: Novozymes Japan Ltd., Chiba, Japan.

2: Novozymes (China) Investment Co. Ltd., Beijing, China

3: Graduate School of Agriculture, Kyoto University, Kyoto, Japan

**Corresponding author:**

Shinobu Takagi

Tel.: +81 80 6739 8716

e-mail: s-takagi3@outlook.jp

Supplemental data

**atagggagaaaaaccgagacaacgatggaactcccatgtagattccaccgccccaattactgttttgggcaatcctgttgataagacgcattctagagttgtttcatgaaagggttacgggtgttgattggtttgagatatgccagaggacagatcaatctgtggtttgctaaactggaagtctggtaaggactctagcaagtccgttactcaaaaagtcataccaagtaagattacgtaacacctgggcatgactttctaagttagcaagtcaccaagagggtcctatttaacgtttggcggtatctgaaacacaagacttgcctatcccatagtacatcatattacctgtcaagctatgctaccccacagaaataccccaaaagttgaagtgaaaaaatgaaaattactggtaacttcaccccataacaaacttaataatttctgtagccaatgaaagtaaaccccattcaatgttccgagatttagtatacttgcccctataagaaacgaaggatttcagcttccttaccccatgaacagaaatcttccatttaccccccactggagagatccgcccaaacgaacagataatagaaaaaagaaattcggacaaatagaacactttctcagccaattaaagtcattccatgcactccctttagctgccgttccatccctttgttgagcaacaccatcgttagccagtacgaaagaggaaacttaaccgataccttggagaaatctaaggcgcgaatgagtttagcctagatatccttagtgaagggttgttccgatacttctccacattcagtcatagatgggcagctttgttatcatgaagagacggaaacgggcattaagggttaaccgccaaattatataaagacaacatgtccccagtttaaagtttttctttcctattcttgtatcctgagtgaccgttgtgtttaatataacaagttcgttttaacttaagaccaaaaccagttacaacaaattataacccctctaaacactaaagttcactcttatcaaactatcaaacatcaaaa**ATGGCTAGAATTCCAAAAGCAGTATCGACACAAGATGACATTCATGAATTGGTCATCAAAACCTTCCGTTGTTACGTTCTCGACTTAGTCGAACAGTATGGTGGTGGTCACCCTGGTTCTGCCATGGGTATGGTCGCCATTGGTATCGCTCTGTGGAAGTACCAGATGAAGTACGCTCCAAATGATCCAGACTACTTCAACAGAGATCGTTTTGTCTTGTCAAACGGTCACGTCTGTCTGTTCCAATACTTGTTCCAGCACTTAACTGGTTTGAAGGAGATGACTGTCAAGCAACTTCAATCTTACCACTCTTCCGATTATCACTCATTGACTCCTGGACACCCTGAAATTGAGAACCCTGCTGTTGAGGTTACCACTGGTCCCCTGGGACAAGGTATCTCTAACGCTGTCGGTATGGCCATTGGTTCAAAGAACCTGGCCGCTACTTACAACAGACCTGGCTTCCCTGTCGTTGACAACACTATCTATGCTATTGTTGGTGATGCTTGTTTGCAAGAGGGACCTGCTTTGGAATCGATTTCCTTAGCCGGTCACTTGGCCTTGGACAACCTTATTGTGATCTACGACAACAACCAGGTTTGTTGTGATGGTTCCGTCGATGTTAACAACACCGAAGACATCTCCGCAAAGTTCAGAGCTCAGAACTGGAATGTTATCGACATTGTAGACGGTTCTAGAGATGTCGCTACCATTGTCAAGGCTATCGATTGGGCCAAGGCTGAGACTGAGAGACCAACTCTGATCAACGTTAGAACTGAAATTGGACAGGATTCTGCTTTCGGTAACCACCACGCTGCTCACGGTTCTGCTCTAGGTGAGGAAGGTATCCGGGAGTTGAAGACTAAGTACGGTTTTAACCCTGCCCAAAAGTTCTGGTTCCCTAAAGAAGTATACGACTTCTTTGCTGAGAAACCAGCTAAAGGTGACGAGTTAGTAAAGAACTGGAAAAAGTTAGTTGATAGCTATGTCAAAGAGTACCCTCGTGAGGGACAAGAGTTCCTTTCTCGTGTTAGAGGTGAGCTTCCAAAGAACTGGAGAACTTACATTCCTCAAGACAAGCCTACCGAACCAACCGCCACCAGAACCTCTGCTAGAGAAATTGTTAGGGCCCTTGGAAAGAACCTTCCTCAAGTTATTGCCGGTTCCGGTGACTTATCTGTCTCAATTCTTTTGAACTGGGACGGAGTGAAGTACTTCTTCAACCCTAAGTTACAGACTTTCTGTGGATTAGGTGGTGACTACTCTGGTAGATATATTGAGTTTGGTATCAGAGAACACTCTATGTGTGCTATTGCCAACGGTTTGGCTGCATACAACAAGGGTACTTTCTTGCCTATTACCTCTACCTTCTACATGTTCTACCTGTATGCAGCACCTGCCTTGCGTATGGCTGCTCTTCAAGAGTTGAAAGCGATTCACATTGCTACACACGACTCTATTGGAGCTGGTGAAGATGGTCCAACCCACCAGCCTATTGCTTTGTCTTCATTATTCAGAGCTATGCCCAACTTCTACTACATGAGACCAGCCGATGCTACCGAAGTTGCAGCTCTGTTTGAAGTGGCTGTTGAGCTTGAACACTCCACATTGCTTTCTCTGTCCAGACACGAGGTTGACCAATACCCAGGTAAGACTTCTGCCCAAGGAGCCAAAAGAGGTGGTTACGTTGTTGAAGACTGCGAAGGAAAGCCAGATGTGCAACTGATCGGAACTGGTTCCGAGTTGGAATTCGCTATTAAGACTGCTCGTTTGCTAAGACAACAGAAGGGATGGAAGGTCAGAGTTCTGTCATTCCCATGTCAGAGATTGTTTGACGAGCAGTCTATTACTTACAGACGTTCCGTCCTTAGAAGAGGAGAAGTTCCAACTGTCGTTGTTGAGGCCTATGTCGCATACGGATGGGAGAGATACGCCACTGCTGGTTACACCATGAACACCTTCGGTAAGTCTCTTCCTGTTGAGGATGTCTACAAATACTTCGGATACACTCCTGAGAAGATTGGTGAGAGAGTGGTTCAATATGTCAACTCTATCAAGGCTAGTCCTCAAATCCTTTACGAATTCCACGACTTGAAGGGAAAACCAAAGCATGACAAGTTGTAA (3179 bases)

Fig.S1. Sequence of *DAS1* gene of *Komagataella phaffii* (*Pichia pastoris*) identified from genome-mining. Sequence of lowercase in bold is 5’-upstream regions of 1-kb used as *DAS1* promoter. Sequence of uppercase is encoding region of *DAS1* gene. Whole sequence is identical to PAS-Chr3-0832 (*DAS1* homologue) including 5’-upstream, at 1608584-1611762 (-strand) of chromosome 3 of *P. pastoris* (*K. phaffii*) GS115 (GenBank NC_012965).


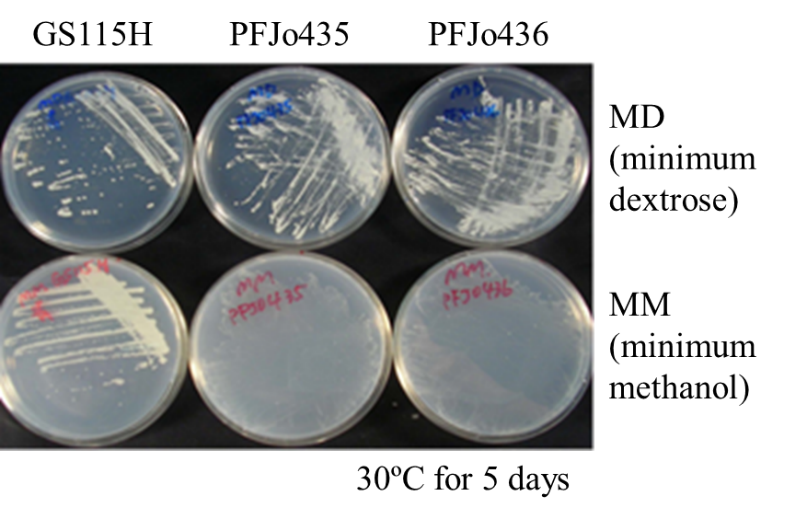


Fig.S2. Growth of *KpTRM1* deletants, PFJo435 and PFJo436, derived from GS115 on methanol plate. PFJo435 is described in US 8.236,528 B2 (1). GS115H is a derivative of GS115 of which *HIS4* gene was complemented by wild type *HIS4*.

**(A)**


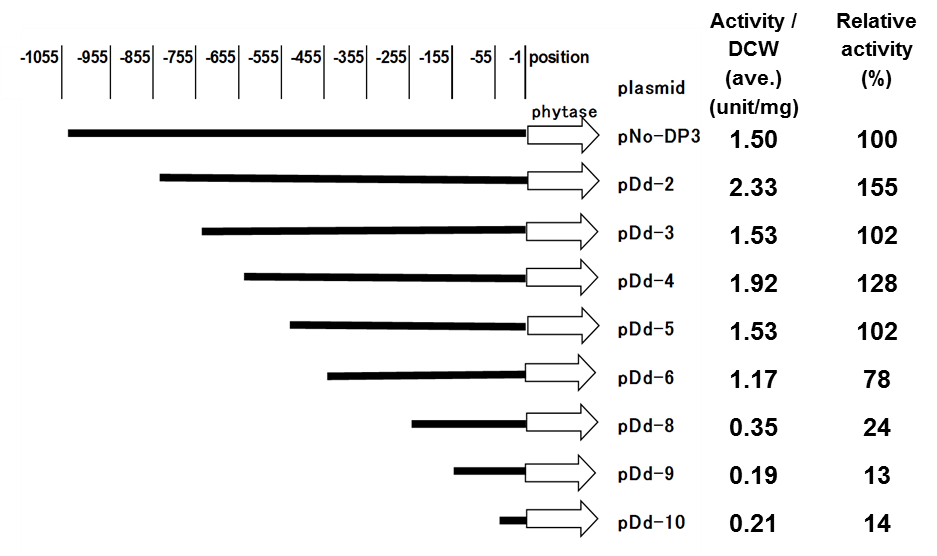


**(B)**


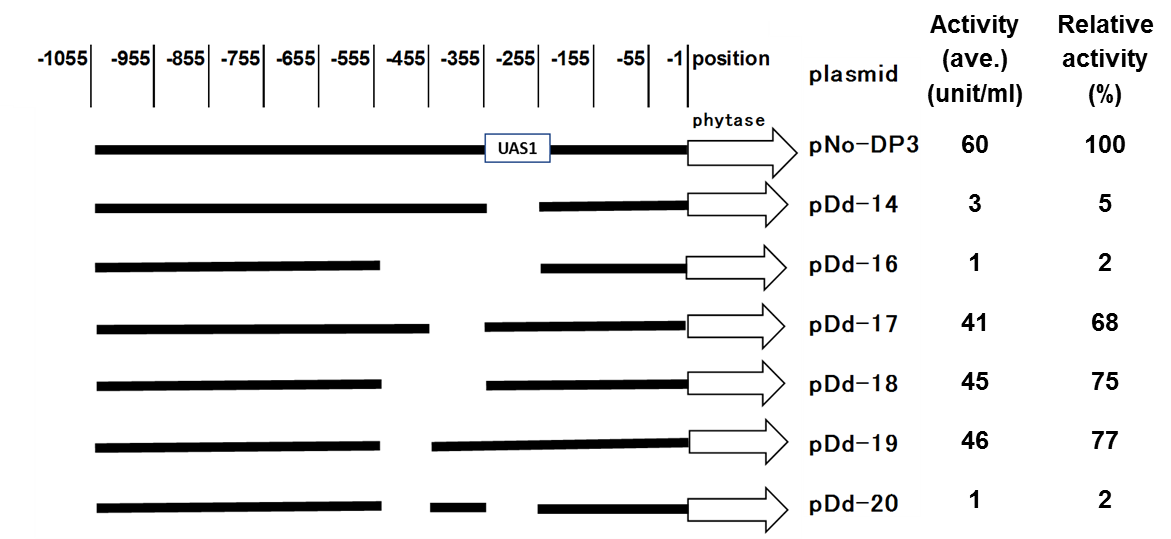


FIG. S3. Deletion analysis of *DAS1* promoter and screening for UAS for methanol induction (2). (A) Structure of deletion variants of different lengths of *DAS1* promoter and phytase activity in shaking flasks when using GS115 as the host strain. Deletion of the region between -555 ~ -255 significantly reduced the activity. (B) Further deletion analysis to screen UAS. The regions in blank were the regions deleted in each plasmid. Tested conditions were the same as (A). The region between -355 ~ -255 was found to be important for phytase expression with methanol, and termed UAS1_DAS1_. A part of original data, Relative activity (%), was described in PCT patent application WO2010/004042 (2).

Table S1. Yeast strains used in this study

| Strain | Genotype | Phenotype* | Source or reference |
| --- | --- | --- | --- |
| KM71 | *his4, aox1*::*ARG4, arg4* | His^-^ Mut^S^ | Invitrogen |
| AOX3 | KM71 *aox1*::pPIC9K-Cb (P*_AOX1_*-native phytase -*HIS4*) | His^+^ Mut^S^ Phy | This study |
| AOX36 | KM71 *aox1*::pPICNoT-G01651 (P*_AOX1_*-syn.phytase -*HIS4*) | His^+^ Mut^S^ Phy | This study |
| GS115 | *his4* | His^-^, Mut^+^ | Invitrogen |
| AOX94 (A94) | GS115 *aox1*::pPICNoT-G01651 (P*_AOX1_*-syn.phytase -*HIS4*) | His^+^ Mut^S^ Phy | (1) |
| GAP46H | GS115, pGAPa-G01651 (P*_GAP_*- syn.phytase - Zeocin), *his4*::pPIC9K (*HIS4*) | His^+^ Mut^+^ Phy Zeocin^r^ | This study |
| DAS40 | GS115, pNo-DP3 (P*_DAS1_*-syn.phytase - *HIS4*) | His^+^ Mut^+^ Phy | (1) |
| DPrm11 | DAS40, pGPrm (P*_GAP_*-*KpTRM1*- Zeocin) | His^+^ Mut^+^ Phy Zeocin^r^ | This study |
| 2-3 | GS115 *his4*::pDd-2 (P*_DAS1’_* -syn.phytase - *HIS4*) | His^+^ Mut^+^ Phy | This study |
| 28-2 | GS115 *his4*::pDd-28 (P*_DAS1’+3xUAS_* - syn.phytase - *HIS4*) | His^+^ Mut^+^ Phy | This study |
| 2P-4 | 2-3, pGPrm (P*_GAP_*-*KpTRM1*- Zeocin) | His^+^ Mut^+^ Phy Zeocin^r^ | This study |
| 28P-14 | 28-2, pGPrm (P*_GAP_*-*KpTRM1*- Zeocin) | His^+^ Mut^+^ Phy Zeocin^r^ | This study |

*Mut^S^: methanol utilization slow, Mut^+^: methanol utilization plus (positive), Phy: expressing phytase under defined condition

Table S2. Plasmids used in this study

| Plasmid | Description | Source or reference |
| --- | --- | --- |
| pIC9K | *HIS4*; P*_AOX1_*-based expression vector | Invitrogen |
| pPIC-NoT | pPIC9K based expression vector, α-factor signal sequence deleted | (1) |
| pPICNoT-G01651 | Synthetic gene of *C. braakii* phytase fused to *S. cerevisiae* α-factor signal sequence was ligated into *Bam*HI/*Eco*RI sites of pPIC-NoT to generate P*_AOX1_*- syn,phytase | (1) |
| pNo-DP3 | 1.0-kb upstream region of *DAS1* gene was ligated at *Nsi*I/*Nde*I sites of pPICNoT-G01651 to replace P*_AOX1_* with P*_DAS1_* and generate P*_DAS1_*-syn.phytase | (1) |
| pDd-2 | Upstream region of *DAS1* gene of pNo-DP3 was replaced with a shorter version of P*_DAS1’_* by In-Fusion | (2) |
| pDd-28 | Three copies of UAS1_DAS1_ was fused to the upstream of P*_DAS1’_* of pDd-2 | (2) |
| pGAPZαA | Zeocin^R^; P*_GAP_*-based expression vector | Invitrogen |
| pGAPα-G01651 | Synthetic gene of *C. braakii* phytase fused to *S. cerevisiae* α-factor signal sequence was ligated into *Bst*BI/*Eco*RI sites of pGAPZαA to generate P*_GAP_*-syn. phytase | This study |
| pGPrm | *KpTRM1* gene was ligated at *Bst*BI/*Sal*I sites of pGAPZαA (P*_GAP_*-PpTRM1) | (1) |
| pGMxr | *MXR1* gene was ligated at *Bst*BI/*Xba*I sites of pGAPZaA (P*_GAP_*-MXR1) | (1) |

**Reference**

1. Takagi, S., Tsutsumi, N., Terui, Y., Kong, X.Y. Method for methanol independent induction from methanol inducible promoters in *Pichia*. US 8.236,528 B2 Novozymes A/S, (2012)
2. Tsutsumi, N., Takagi, S. *Pichia pastoris* *DAS1* promoter variant. PCT application WO 2010/004042, Novozymes A/S (2010)
